# Predictors of skin and soft tissue infections among sample of rural residents who inject drugs

**DOI:** 10.1186/s12954-020-00447-3

**Published:** 2020-12-02

**Authors:** Amelia Baltes, Wajiha Akhtar, Jen Birstler, Heidi Olson-Streed, Kellene Eagen, David Seal, Ryan Westergaard, Randall Brown

**Affiliations:** 1 Department of Family Medicine and Community Health, School of Medicine and Public Health, University of Wisconsin, Madison, WI USA; 2 Department of Medicine, School of Medicine and Public Health, University of Wisconsin, Madison, WI USA; 3 Department of Biostatistics and Medical Informatics, School of Medicine and Public Health, University of Wisconsin, Madison, WI USA; 4 Wisconsin Department of Health Services Hepatitis C Program, Madison, USA; 5 School of Public Health and Tropical Disease, Tulane University, New Orleans, USA

**Keywords:** Skin and soft tissue infections, People who inject drugs, Heroin, Opioid, Methamphetamine, Injection drug use, Public health

## Abstract

**Introduction:**

Skin and soft tissue infections (SSTIs) are among the leading causes of morbidity and mortality for people who inject drugs (PWID). Studies demonstrate that certain injection practices correlate with SSTI incidence among PWID. The opioid epidemic in the USA has particularly affected rural communities, where access to prevention and treatment presents unique challenges. This study aims to estimate unsafe injection practices among rural-dwelling PWID; assess treatments utilized for injection related SSTIs; and gather data to help reduce the overall risk of injection-related SSTIs.

**Methods:**

Thirteen questions specific to SSTIs and injection practices were added to a larger study assessing unmet health care needs among PWID and were administered at six syringe exchange programs in rural Wisconsin between May and July 2019. SSTI history prevalence was estimated based on infections reported within one-year prior of response and was compared to self-reported demographics and injection practices.

**Results:**

Eighty responses were collected and analyzed. Respondents were white (77.5%), males (60%), between the ages 30 and 39 (42.5%), and have a high school diploma or GED (38.75%). The majority of respondents (77.5%) reported no history of SSTI within the year prior to survey response. Females were over three times more likely to report SSTI history (OR = 3.07, *p* = 0.038) compared to males. Water sources for drug dilution (*p* = 0.093) and frequency of injecting on first attempt (*p* = 0.037), but not proper skin cleaning method (*p* = 0.378), were significantly associated with a history of SSTI. Injecting into skin (*p* = 0.038) or muscle (*p* = 0.001) was significantly associated with a history of SSTI. Injection into veins was not significantly associated with SSTI (*p* = 0.333).

**Conclusion:**

Higher-risk injection practices were common among participants reporting a history of SSTIs in this rural sample. Studies exploring socio-demographic factors influencing risky injection practices and general barriers to safer injection practices to prevent SSTIs are warranted. Dissemination of education materials targeting SSTI prevention and intervention among PWID not in treatment is warranted.

## Background

Rates of injection drug use (IDU) have grown in parallel with the US opioid epidemic since 2000 [9, 12]. IDU is a growing public health concern given the associated risk of the transmission of blood-borne diseases, such as human immunodeficiency virus (HIV), hepatitis C virus (HCV), and of skin and soft tissue infections (SSTIs) [9], [24]. Common IDU-related SSTIs include skin abscesses, cellulitis, osteomyelitis, and infective endocarditis [6]. Bacteria commonly found in skin flora, commonly *Staphylococcus aureus*, may contribute to IDU-related SSTIs when introduced into the subcutaneous space during injection [1, 12]. In people who inject drugs (PWID) with particular constitutional vulnerabilities (e.g., heart valve malformations, septal defects), these potential pathogens, as well as others, may also seed deeper tissues leading to infection nidus, abscess, and/or septic emboli. PWID have demonstrated higher rates of *S. aureus* colonization in skin flora than the general population, that has been posited to potentially predispose them to a greater risk of cellulitis and cutaneous abscess [1]. While *S. aureus* is by far most commonly identified pathogen, identified in > 75% of SSTIs [3], other microorganisms include streptococci, gram-negative bacilli, and fungi [16]. Additionally, a study by Harris et al. found the overuse of acidifiers, such as citric acid, in preparing substances to be associated with increased odds of prolonged SSTI and inflammation (Harris et al. 2019). Venous damage as a result of the use of acidifiers can lead to increased risk of IDU-related SSTI development (Harris et al. 2019, Ciccarone and Harris 2015).

Infectious diseases among people who use drugs are associated with a variety of social determinants and behavioral factors (Galea and Vlahov 2002). Infections associated with IDU disproportionately affect non-Hispanic white Americans experiencing economic distress and populations living in rural and suburban areas [9], [24]. Sociocultural factors surrounding injection drug use place females at a higher risk of developing SSTIs, such as relying on other individuals to prepare their drug, increased potential for shared injection equipment; and greater difficulty finding veins [4, 14, 26, 29, 32]. Individuals recently experiencing homelessness are also more likely to report a history of SSTI, potentially relating to injecting in unsafe or unsanitary environments [4, 14, 26].

Behavioral factors, such as high risk injection practices, are commonly associated with SSTIs among PWID [26], Galea and Vlahov (2002). Using and sharing contaminated single-use syringes, needles, and ancillary equipment places individuals at higher risk of spreading infectious agents; prior HIV and HCV outbreaks throughout the USA have been closely associated with the sharing of such equipment [14, 23, 24], Galea and Vlahov 2002). In addition to non-sterile injection equipment, previous results suggest water sources for drug dilution also play a role in the transmission of blood-borne diseases and bacterial infections [22]. A lack of proper skin cleaning technique, such as using alcohol wipes, also confers higher SSTI risk [20]. The type of injection has also been shown as a major risk factor associated with abscess formation, and ‘skin popping’ (subcutaneous injecting) and ‘muscling’ (intramuscular injecting) are highly associated with SSTI development [15, 25, 32]. Similarly, individuals injecting in their hands, groin, or legs are also at higher risk of developing an infection (Hope et al. [15]. Regardless of the location of injection, repeated injection attempts at the same anatomical site increases overall risk of tissue and venous damage and bacterial inoculation [29].

Several studies have demonstrated the varying risks of injecting different illicit substances. Injecting a mixture of heroin and cocaine, otherwise known as ‘speedball,’ puts individuals at a higher risk of developing abscesses; this is thought to be due to the synergistic effects of local irritation and tissue vasoconstriction [15, 29], Galea and Vlahov 2002; Lloyd-Smith et al. 2008). The use of methamphetamine is a growing concern throughout the USA, with nearly sixfold increases in the presence of methamphetamine found in urine drug samples [33]. Additionally, methamphetamine use has increased since 2015 among people reporting any opioid use, with threefold increases being seen among people reporting heroin use [30]. Chronic methamphetamine use is associated with a higher risk of contracting infections, and individuals who use methamphetamine suffer from altered judgment and reduced inhibitions, potentially provoking them to partake in risky behaviors and thus increasing their overall risk of acquiring bacterial microbes and other opportunistic infections [28].

Hospitalizations to treat IDU-related SSTIs have risen in conjunction with increasing incidence of such infections [18]. Younger populations are at increased risk of hospitalization due to IDU-related infections [18], Nenninger et al. [21]. Between 1998 and 2001 in the USA, Takahashi et al. [31] estimated that there were 106,126 hospitalizations for IDU-related SSTIs, costing an estimated $193 million annually, and these patients were often uninsured or covered by publicly funded insurance programs, such as Medicare or Medicaid [31]. Contrarily, individuals who frequently develop IDU-related SSTIs have been shown to use a variety of self-treatment methods in lieu of seeking professional medical treatment, potentially due to their confidence in self-diagnosis and treatment due to the commonality of such infections [19].

The prevalence of opioid prescriptions and related overdose deaths has increased over the past several years throughout the USA, with rural areas being disproportionately affected; particularly, the Midwest has seen dramatic increases in opioid-related morbidities and mortality rates [8]. In Wisconsin, rural counties facing economic distress report some of the highest opioid overdose rates [27]. In addition to overdose, rural areas may be disproportionately affected by IDU-related SSTI hospitalizations because access to harm reduction services is often limited in these areas. The lack of resources could be preventing PWID from easily accessing safe injection supplies and subsequently increasing their risk of infection [21].

Limited studies have been conducted to understand injection drug use among rural-dwelling individuals. Similarly, infection risk and SSTI treatment practices among this population have yet to be explored in-depth. Given the limited resources and barriers in accessing treatment within these communities, it is important to understand their current practices and needs in order to promote more targeted interventions to decrease the risk of SSTIs. Research among rural counties is merited to determine which unsafe injection practices and substances are closely correlated to history of SSTI and how harm reduction strategies could target this population to more effectively reduce rates of IDU-related SSTIs. This study aims to: (1) estimate unsafe injection practices currently used among PWID residing in rural areas, particularly among those with a history of IDU-related SSTIs; (2) assess treatments utilized for IDU-related SSTIs among rural residents; and (3) gather data that could guide future interventions and educational materials aimed at reducing the overall risk of IDU-related SSTIs.

## Methods

### Institutional review

This study involved human participants and was reviewed by the Institutional Review Board of the University of Wisconsin-Madison. Written informed consent to participate in this study was provided by each participant.

### Survey development

This study is part of a larger study assessing unmet health care needs among rural-dwelling PWID. The one-time, cross-sectional survey used audio computer-assisted self-interview (ACASI) software to assist in ease of administration. Between March and May 2019, a total of 13 questions specific to SSTIs were developed and added to the overall survey, making the survey a total of 211 items. The 211-item survey, which included the SSTI-specific questions, was then administered to participants. These questions were adapted from Phillips and Stein’s [25] Bacterial Infections Risk Scale for Injectors (BIRSI), a 17-item scale aimed at capturing a variety of behavioral practices closely associated with increased risk of SSTIs among PWID [25]. Similarly, the SSTI-specific questions created and adapted for this study focused on behavioral practices related to IDU, including skin-cleaning methods and water sources used for drug dilution. Additionally, information surrounding injection type (e.g., subcutaneous, intravenous, or intramuscular injection) and anatomical location of injection was collected. SSTI prevalence was estimated based on if individuals reported developing an infection within the year prior to responding to the survey. If a respondent cited SSTIs within the year prior to response, treatment methods used for their most recent SSTI were collected.

Pre-existing questions within the overall survey collected additional data surrounding demographics, syringe and needle sharing behaviors, hospitalizations related to SSTIs, and primary drug of choice. Demographics information for survey respondents included: sex, age, race, education level, insurance status, and history of experiencing homelessness. All participants resided in rural communities.

### Recruitment

The larger study recruited a total of 998 participants through Vivent Health, a statewide organization offering a large, multi-site syringe exchange program. This provided an opportunity to survey a geographically disperse population of PWID in rural communities across Northern Wisconsin. Participants were recruited using respondent-driven sampling [13]. Using a respondent-driven sampling approach allowed for the recruitment of individuals who may not utilize the syringe service program and may be at higher risk of adverse outcomes associated with injecting drugs. The larger survey was administered from September 2017 to July 2019 at six different Vivent Health locations in Brown, Douglas, Eau Claire, La Crosse, Outagamie, and Marathon counties in Wisconsin. The revised survey containing the SSTI-specific questions was administered between May and July 2019 and received a total of 80 responses from all six locations. While the larger study sought to enroll a specific number of participants, the SSTI questions were added midway through data collection for the larger study and served as exploratory data. Therefore, a specific sample size was not sought after for responses to the SSTI-related questions.

Eligible respondents were 15 years or older, injected drugs within the 30 days prior to survey response, and resided in a rural community. Eligible participants were directed to their local Vivent Health location to simultaneously fill out the ROI survey and receive rapid HIV, HCV, and syphilis testing. All respondents were compensated $20 in cash for completing the survey and an additional $10 for each peer referred to the study.

### Statistical analysis

Logistic regression models were used to estimate the odds of SSTI based on demographics and injection practices, for categorical variables. Chi-square tests were used for assessing overall associations of SSTI with specific skin cleaning methods and water sources. Mann–Whitney–Wilcoxon tests were used to assess associations with SSTI history and ordered variables. Significance was assessed at the alpha = 0.10 level. SSTI history was defined as a reported SSTI within a year prior to survey response. Analysis was conducted using R version 3.6.3.

## Results

In total, 80 responses to the SSTI-specific questions were collected and analyzed. Shown in Table 1, respondents were primarily Caucasian (77.5%) males (60%) between the ages 30 and 39 (42.5%) who had received a high school diploma or GED (38.75%), received health insurance through Medicaid (70%), and were unstably housed within the 6 months prior to survey response (65%). Respondents most commonly (50%) reported methamphetamine as their primary drug of choice, while 25% of respondents reported heroin. Of the 80 respondents, 62 (77.5%) individuals reported no history of skin and soft tissue infections within the year prior to survey response, while 18 (22.5%) individuals reported a history of SSTI during this same time frame. Females were more likely to report of history of SSTI in comparison with males (61.1% vs 33.9%).

**Table 1:** Demographics

Variable	History of skin and soft tissue infection		Total
	Yes	No	n = 80
	(n = 18)	(n = 62)	n (%)
	n (%)	n (%)	
Sex			
Male	7 (38.9)	41 (66.1)	48 (60.0)
Female	11 (61.1)	21 (33.9)	32 (40.0)
Age			
18–29	4 (22.2)	17 (27.4)	21 (26.3)
30–39	7 (38.9)	27 (43.5)	34 (42.5)
40–49	6 (33.3)	9 (14.5)	15 (18.8)
50–59	1 (5.6)	8 (12.9)	9 (11.3)
60 +	0 (0.0)	1 (1.6)	1 (1.3)
Race			
Caucasian	14 (77.8)	48 (77.4)	62 (77.5)
African American	2 (11.1)	2 (3.2)	4 (5.0)
American Indian	1 (5.6)	10 (16.1)	11 (13.8)
Mixed race	1 (5.6)	2 (3.2)	3 (3.8)
Education			
Less than high school	3 (16.7)	12 (19.4)	15 (18.8)
High school diploma or GED	8 (44.4)	23 (37.1)	31(38.8)
Some college	7 (38.9)	22 (35.5)	29 (36.3)
Associate’s degree	0 (0.0)	4 (6.5)	4 (5.0)
Bachelor’s degree or higher	0 (0.0)	1 (1.6)	1 (1.3)
Homelessness (past 6 months)			
Yes	11 (61.1)	41 (66.1)	52 (65.0)
No	7 (38.9)	21 (33.9)	28 (35.0)
Health insurance			
Private or commercial	0 (0.0)	5 (8.1)	5 (6.3)
Medicaid	9 (50.0)	31 (50.0)	40 (50.0)
Medicaid expansion (ACA)	0 (0.0)	3 (4.8)	3 (3.8)
Medicare	2 (11.1)	0 (0.0)	2 (2.5)
Other	1 (5.6)	1 (1.6)	2 (2.5)
Drug of choice			
Methamphetamine	7 (38.9)	33 (53.2)	40 (50.0)
Heroin	6 (33.3)	14 (22.6)	20 (25.0)
Cocaine	2 (11.1)	3 (4.8)	5 (6.3)
Prescription anxiety drugs	1 (5.6)	4 (6.5)	5 (6.3)
Methadone	1 (5.6)	1 (1.6)	2 (2.5)
Opiate pain killers	0 (0.0)	2 (3.2)	2 (2.5)
Synthetics	0 (0.0)	2 (3.2)	2 (2.5)
Other	1 (5.6)	2 (3.2)	3 (3.8)

Shown in Table 2, respondents reported using a variety of sterile skin cleaning practices prior to injecting, including alcohol pads (60%), soap and water (12.5%), hydrogen peroxide (1.25%), or Purell (1.25%). However, respondents also reported using tap water (3.75%), saliva (2.5%), and never cleaning their skin prior to injecting (1.25%). Participants reported using sterile skin cleaning practices (i.e., alcohol pad, hydrogen peroxide, hand sanitizer, or soap and water) were no less likely to report a history of SSTIs in comparison with those who did not (i.e., never cleaning skin prior to injection, saliva, or tap water alone) (*p* = 0.378) (Table 2). Respondents citing less frequent hand washing prior to injection had no increased odds of reporting a history of SSTIs (*p* = 0.200). Water sources for drug dilution bore a significant relationship with SSTIs, and individuals using purified sources of water—such as water purchased from a store or sterile water obtained from syringe exchange programs—were more likely to report a history of SSTIs in comparison with those who used non-purified sources of water—such as tap water from either their home or a public bathroom sink (*p* = 0.093). No respondents reported the use of non-sterile water from a public or private toilet.

**Table 2:** Behavioral injection practices

Variable		History of skin and soft tissue infection		*p* value	Odds ratio
		Yes (n = 18)	No (n = 62)		
		n (%)	n (%)		
Clean skin prior to injection	Always	4 (22.2)	14 (22.6)	0.667	Ref
	Most of the time	4 (22.2)	13 (21.0)		1.09 each decrease in freq
	Sometimes	3 (16.7)	14 (22.6)		
	Rarely	3 (16.7)	10 (16.1)		
	Never	4 (22.2)	9 (14.5)		
Skin cleaning method	Alcohol pad	8 (44.4)	40 (64.5)	0.073*	
	Soap and water	2 (11.1)	8 (12.9)		
	Tap water	1 (5.6)	2 (3.2)		
	Saliva	0 (0.0)	2 (3.2)		
	Hydrogen peroxide	1 (5.6)	0 (0.0)		
	Purell	0 (0.0)	1 (1.6)		
	Never clean before injecting	1 (5.6)	0 (0.0)		
	Prefer not to answer	1 (5.6)	0 (0.0)		
Skin cleaning method	Sterile methods†	11	49	0.378	Ref
	Unsterile methods‡	2	4		2.23
Wash hands prior to injection	Always	4 (22.2)	16 (25.8)	0.205	Ref
	Most of the time	1 (5.6)	15 (24.2)		1.29 each decrease in freq
	Sometimes	5 (27.8)	11 (17.7)		
	Rarely	5 (27.8)	11 (17.7)		
	Never	3 (16.7)	7 (11.3)		
Water source	Tap water (home)	12 (66.7)	46 (74.2)	0.559	
	Purchase from store	3 (16.7)	4 (6.5)		
	Syringe exchange	2 (11.1)	3 (4.8)		
	Tap water	0 (0.0)	4 (6.5)		
	(public bathroom)				
	N/A, liquid drug	0 (0.0)	2 (3.2)		
	I don’t know	0 (0.0)	1 (1.6)		
	Other	1 (5.6)	2 (3.2)		
Water source	Sterile sources§	5	7	0.093*	Ref
	Unsterile sources#	12	50		0.34

^*^Significant on the 0.10 level

^†^Sterile skin methods: alcohol pad, hydrogen peroxide, hand sanitizer, soap and water

^‡^Unsterile skin methods: never clean skin before injective, saliva, tap water

^§^Sterile water sources: purchase from store, syringe exchange program

^#^Unsterile water sources: tap water (home), tap water (public bathroom)

Regarding injection site Table 3, individuals reporting ‘skin popping’ (subcutaneous injecting) (6.25%, *p* = 0.038, OR = 6.00) and ‘muscling’ (intramuscular injecting) (6.25%, *p* = 0.001, OR = 17.4) were significantly more likely to report a history of SSTI in comparison with those injecting intravenously (93.7%, *p* = 0.333, OR = 0.41). Individuals reported injecting into various anatomical locations, including cubita fossa (42.5%), forearm (32.5%), hand (8.75%), and upper arm (7.5%). The anatomical location of injection was not significantly correlated with a history of SSTI (*p* = 0.831). Individuals able to inject on their first attempt more frequently (e.g., always or most of the time) were significantly less likely to report a history of SSTI (*p* = 0.037). Frequency of injection—whether daily, weekly, or monthly—did not exhibit significance in relation to SSTI prevalence (*p* = 0.892).

**Table 3:** Injection sites

Variable		History of skin and soft tissue infection		*p* value	Odds ratio
		Yes (n = 18)	No (n = 62)		
		n (%)	n (%)		
Find vein on first injection attempt	Always	3 (16.7)	23 (37.1)	0.037**	Ref
	Most of the time	8 (44.4)	25 (40.3)		1.66 each decrease in freq
	Sometimes	1 (5.6)	7 (11.3)		
	Rarely	4 (22.2)	4 (6.5)		
	Never	2 (11.1)	2 (3.2)		
Anatomical injection location	Cubital fossa	8 (44.4)	26 (41.9)	0.831 (overall)	Ref
	Forearm	4 (22.2)	22 (35.5)		0.59
	Hand	2 (11.1)	5 (8.1)		1.3
	Upper arm	2 (11.1)	4 (6.5)		1.3
	Other	2 (11.1)	5 (8.1)		1.62
Injection type (multiple selection)	Vein	16 (88.9)	59 (93.8)	0.333	0.41
	Skin	3 (16.7)	2 (3.2)	0.038**	6
	Muscle	4 (22.2)	1 (1.6)	0.001**	17.4
	I don’t know	1 (5.6)	0 (0.0)	0.062*	
	Prefer not to answer	0 (0.0)	1 (1.6)	0.588	
Injection frequency	> 3 × daily	5 (27.8)	11 (17.7)	0.892	
	2–3 × daily	3 (16.7)	15 (24.2)		
	Daily	1 (5.6)	8 (12.9)		
	> Weekly	3 (16.7)	11 (17.7)		
	Weekly	1 (5.6)	7 (11.3)		
	> Once in past 30 days	2 (11.1)	5 (8.1)		
	Once in past 30 days	2 (11.1)	4 (6.5)		

^*^Significant on the 0.10 level

^**^Significant on the 0.05 level

Individuals reporting a history of SSTI (n = 18, 22.5%) within the year prior to survey response reported a variety of treatment methods (Table 4). At-home remedies for SSTIs included warm compresses (56%), the use of over-the-counter medications (22.2%), and self-drainage of the abscess outside of a clinical setting (27.8%). However, individuals also sought out a range of professional medical care for the treatment of their SSTIs. Professional medical resources included going to an emergency room or urgent care (55.5%), using prescribed medications (33.3%), having either a procedure in the emergency room (11.1%) or surgical intervention (11.1%), or requiring an inpatient hospital stay (5.5%).

**Table 4:** SSTI treatment methods

	Treatment method (s) utilized	Total n = 18 n (%)
Medical treatments	Emergency room or urgent care visit	10 (55.6)
	Prescribed medications	6 (33.3)
	Procedure in emergency room or urgent care	2 (11.1)
	Surgery in operating room	2 (11.1)
	Inpatient hospital stay	1 (5.6)
At-home remedies	Warm compress at home	10 (55.6)
	Over the counter medications	4 (22.2)
	Drained abscess at home	3 (16.7)

## Discussion

Primary findings of the described study indicate that participants reporting a history of SSTIs in the prior year were likely to partake in a variety of unsafe injection practices. While skin cleaning methods prior to injection did not exhibit significant correlations with a history of IDU-related SSTIs, the type of water sources used for drug dilution did. Females are up to three times more likely to report a history of SSTIs in comparison with males, leading us to believe that there may be sociocultural and geographic factors in the rural setting contributing to such findings. Although the anatomical location of injection did not bear a significant correlation to SSTI prevalence, the type of injection—such as ‘skin-popping’ (subcutaneous), ‘muscling’ (intramuscular), or intravenous—demonstrated significance; individuals reporting subcutaneous and intramuscular injection practices were significantly more likely to report a history of SSTIs compared to those injecting intravenously. Additionally, individuals able to regularly inject on their first attempt (e.g., always or most of the time) were significantly less likely to report a history of SSTIs. Lastly, individuals reporting a history of IDU-related SSTIs within the year prior to survey response cited several treatment methods, including both at-home remedies and professional medical care.

A number of studies have investigated and found similar trends of unsafe injection practices correlating to higher risk of SSTIs among PWID; however, many of these studies are in the context of urban or suburban populations, rather than in rural settings. Similar to our study, several others investigating urban samples have reported higher incidence of IDU-related SSTIs among females [4, 14, 29]. Tuchman [32] found that urban females were more likely to initiate their injection drug use after being influenced by other females and commonly relied on others to inject for them, citing inability to inject themselves and difficulty finding an injection site as primary factors driving their decision [32]. Such circumstances increase their risk of utilizing unsafe injection practices and may lend to the higher rates of SSTIs among females who inject drugs. In the context of rural populations, the gender composition of and interactions among social networks may differ in comparison with urban settings. Cultural norms among rural populations are also different than those in urban areas, potentially serving as additional factors lending to higher rates of SSTIs among females in comparison with males in rural settings.

Site of injection has also been cited as a common factor associated with IDU-related SSTIs. Similar to our findings, Smith et al. [29], Phillips et al. [26], and Murphy et al. [20] revealed ‘skin-popping’ (subcutaneous injection) as a common risk factor associated with IDU-related SSTIs in urban populations [20, 26, 29]. Our findings among PWID in rural areas are consistent with their urban peers, suggesting that both populations use higher-risk sites via either ‘skin-popping’ or ‘muscling’ for injection practices. It should be kept in mind that the extent to which overall duration of injection use, and related health vulnerabilities, might be a contributing, or even driving, factor for this finding is a remaining question in the context of interpreting results of this cross-sectional study (i.e., those who have injected over longer periods of time may have greater challenges locating a viable vein for injection use).

Those citing a history of SSTIs reported the use of a variety of both professional medical treatments and at-home remedies. These findings are consistent with existing literature surrounding urban populations. Monteiro et al. [19] described an urban population of PWID often utilizing emergency medical services to treat SSTIs, while also reporting the use of at-home abscess drainage by non-medical personnel [19]. Although findings are similar between urban and rural populations, individuals residing in rural areas often experience a variety of health-related disparities that may serve as barriers to accessing proper medical care [5]. Among other factors, obstacles in accessing adequate health care in rural areas and stigma attached to seeking care for an injection-related health issue may contribute to the use of at-home remedies for the treatment of IDU-related SSTIs rather than seeking medical treatment.

Similar to our findings, Wright et al. (2020) found no significant relationship between skin cleaning prior to injection and SSTI history among a larger urban population, insinuating that skin cleaning may not serve as a strong protective factor against infection (Wright et al. 2020). However, given that our results present skin cleaning methods used at the time of survey response, rather than the skin cleaning methods used at the time of infection, these results may be indicative of changes in skin cleaning behaviors among PWID following infection in order to help prevent the development of further SSTIs. Contrarily, several studies suggest that skin cleaning methods are a significant predictor within urban populations. Smith et al. [29] and Murphy et al. [20] both present the use of alcohol for skin cleaning prior to injection as a significant protective factor against IDU-related SSTIs in urban populations [20, 29]. Similarly, Phillips and Stein [25] cited infrequent skin cleaning prior to injection as a common risk factor associated with IDU-related SSTIs among an urban population [25]. Urban and rural populations may be impacted differently by skin cleaning practices despite partaking in similar techniques. Rural populations often experience several barriers when accessing syringe exchange programs [5]. Such barriers may place rural populations at a disadvantage for obtaining skin cleaning supplies, such as alcohol wipes, in comparison with individuals residing in urban areas. This may contribute to the differences in skin cleaning significance in the context of IDU-related SSTIs between urban and rural populations who inject drugs. However, based on the contradictory findings between several studies, further research is needed to determine the role of skin cleaning methods and their effectiveness in preventing the development of SSTIs.

Contrary to our results, several studies have found that the anatomical location of injection is significantly correlated with risk of infection. Although our results suggest that individuals injecting into their hands and upper arms were slightly more likely to report a history of SSTI in comparison with individuals injecting into their cubital fossa, there was no significant correlation found. Conversely, past literature surrounding urban samples demonstrates that injection into either upper and lower extremities or the groin region corresponds with history of infection [6, 14].

Additionally, the majority of our sample cited the use of methamphetamine as their primary drug of choice. Although our study found no significant correlation between primary drug of choice and history of SSTI, both Murphy et al. [20] and Phillips and Stein [25] reported the use of ‘speedball,’ or heroin mixed with cocaine, to be commonly associated with IDU-related SSTIs in urban populations [20, 25]. Access to and preference of substances may differ between urban and rural populations, potentially lending to the differences between infections related to the substance injected.

Lastly, our study presents water source for drug dilution—whether sterile or unsterile—as a significant risk factor associated with reporting a history of SSTIs among this rural population. Although significance is not reported, Phillips and Stein’s [25] findings suggest that PWID in urban populations also use a combination of sterile and non-sterile water sources to inject their drugs [25]. Likewise, despite respondents not reporting the use of public or private toilet water among this sample, Harris et al. (2020) report the use of toilet and puddle water or water alternatives, such as cola or lemon juice, used for injecting, which may further lend to risk of SSTI (Harris et al. 2020). Such results indicate that both rural and urban populations partake in risk behaviors associated with water sources and both populations are at risk of developing SSTIs related to their water sources and usage. Further investigation is required to determine the specific relationship between SSTI risk and water source, because although unsterile water sources lend to overall risk of SSTI, individuals with a history of SSTI may also be more likely to seek out sterile water sources to help prevent related infections.

Overall, higher-risk injection practices were common among participants reporting a history of SSTIs living in rural areas. These results are similar to comparative studies conducted among urban and suburban populations. These findings suggest that educational materials targeting PWID not in treatment should encompass a variety of injection behaviors—including ‘skin-popping’ (subcutaneous injection) or ‘muscling’ (intramuscular injection); proper skin cleaning practices; and the use of sterile water sources for drug dilution. Future studies should aim to understand social determinants and cultural factors, such as social networks, present in rural populations which may influence risky injection practices and the general barriers of safer injection practices to prevent SSTIs. With females being up to three times more likely to report a history of infection, future studies should investigate risk factors that are unique to females who inject drugs in rural communities. Given the increasing prevalence of methamphetamine, future research should also focus on the differences in injection practices between people who use methamphetamine as their primary drug of choice versus heroin or cocaine.

Urban hospitals and clinical practices witness high rates of emergency department visits and hospitalizations related to infectious comorbidities associated with injection drug use [31]. Our results suggest that rural populations partake in similar risky behaviors associated with developing bacterial infections and seek out similar medical and non-medical treatments for SSTIs as urban populations. However, rural-dwelling individuals may experience more frequent barriers when attempting to access proper health care services [34]. This places rural populations at a high risk of experiencing life-threatening sequelae. Therefore, rural hospitals should consider partnering with outreach organizations and/or pharmacies to target PWID with better wound education, safer injection practices, and education regarding when it is safe to treat their infections at home versus when to seek medical treatment. By introducing such proactive intervention and prevention methods in both the clinical and public health setting, hospitals and clinical practices will potentially see decreases in emergency department visits and hospitalizations associated with infectious comorbidities related to injection drug use.

### Public health implications

The opioid epidemic is one of the most pressing public health crises of our time. Co-morbidities associated with injection drug use, namely skin and soft tissue infections, place a large financial and care burden on public health agencies and clinical settings [31]. Syringe exchange programs have the potential to serve as a cornerstone in preventing and minimizing the effects of SSTIs as they relate to IDU. Given their expansive coverage across networks of people who inject drugs, syringe exchange programs should consider implementing or expanding existing early prevention and education materials specifically targeting risky injection practices as they relate to SSTIs. Additionally, these public health programs should consider providing resources on proper treatment methods and medical resources for individuals who have already developed an infection. Such prevention materials are one of the first lines of defense in combatting infectious co-morbidities associated with injection drug use and have the potential to make a widespread impact on both populations of people who inject drugs and the notable workload placed on hospitals to care for such patients.

### Limitations

This study should be considered in the context of several limitations: (1) the data presented was collected using a one-time cross-sectional approach, potentially introducing recall bias; (2) the prevalence of skin and soft tissue infections among survey respondents was estimated based self-reported infections within the year prior to survey response and could be skewed; (3) the sample size was relatively small, with all respondents being clients of Vivent Health’s statewide syringe exchange program and could impact power; and (4) the use of respondent driven sampling may underestimate the variability within populations because of the tendency of participants recruiting others with similar characteristics.

## Data Availability

The datasets generated for this study are not currently publicly available at the time of publication.

## Abbreviations

ACASI: Audio computer-assisted self-interview
HCV: Hepatitis C virus
HIV: Human immunodeficiency virus
IDU: Injection drug use
PWID: People who inject drugs
SSTI: Skin and soft tissue infection
